# Biomaterial-Based CRISPR/Cas9 Delivery Systems for Tumor Treatment

**DOI:** 10.34133/bmr.0023

**Published:** 2024-04-30

**Authors:** Mengmeng Li, Fenglei Chen, Qian Yang, Qinglai Tang, Zian Xiao, Xinying Tong, Ying Zhang, Lanjie Lei, Shisheng Li

**Affiliations:** ^1^ Department of Otorhinolaryngology Head and Neck Surgery, the Second Xiangya Hospital, Central South University, Changsha 410011, Hunan, China.; ^2^ College of Veterinary Medicine, Co-innovation Center for Prevention and Control of Important Animal Infectious Diseases and Zoonoses, Yangzhou University, Yangzhou 225009, China.; ^3^ Department of Hemodialysis, the Second Xiangya Hospital, Central South University, Changsha 410011, Hunan, China.; ^4^ Institute of Translational Medicine, Zhejiang Shuren University, Hangzhou 310015, Zhejiang, China.

## Abstract

CRISPR/Cas9 gene editing technology is characterized by high specificity and efficiency, and has been applied to the treatment of human diseases, especially tumors involving multiple genetic modifications. However, the clinical application of CRISPR/Cas9 still faces some major challenges, the most urgent of which is the development of optimized delivery vectors. Biomaterials are currently the best choice for use in CRISPR/Cas9 delivery vectors owing to their tunability, biocompatibility, and efficiency. As research on biomaterial vectors continues to progress, hope for the application of the CRISPR/Cas9 system for clinical oncology therapy builds. In this review, we first detail the CRISPR/Cas9 system and its potential applications in tumor therapy. Then, we introduce the different delivery forms and compare the physical, viral, and non-viral vectors. In addition, we analyze the characteristics of different types of biomaterial vectors. We further review recent research progress in the use of biomaterials as vectors for CRISPR/Cas9 delivery to treat specific tumors. Finally, we summarize the shortcomings and prospects of biomaterial-based CRISPR/Cas9 delivery systems.

## Introduction

Oncological diseases are a prevalent factor that threatens the health and quality of life of people around the world. The number of new cancer cases is increasing year by year, and the mortality rate of most cancers remains high [1]. Commonly used tumor treatment methods include surgical resection, radiotherapy, chemotherapy, and comprehensive treatment. Although the overall survival rate of patients after treatment has improved to some extent, conventional treatments are often accompanied by problems such as chemoradiotherapy resistance, high recurrence rates, and serious adverse effects [2]. As research has progressed, we have discovered that tumor cells possess a variety of genetic changes, and many genetic mutations are prerequisites for driving the malignant development of tumors and drug resistance [3]. Therefore, identification, correction, and/or deletion of such mutated genes are important for the future development of tumor therapies. High-throughput functional screening technologies have provided important genetic insights for oncology research. High-throughput functional gene screening is based on gene editing technology to screen for target phenotypes by interfering with gene function on a genome-wide scale. Holm et al. have implemented a BabySeq program using high-throughput functional screening techniques. High-throughput sequencing was performed on 127 general neonates and 32 patients in the intensive care unit. They found that 9.4% of newborns were at risk for childhood disease; that 3.5% had pathogenic variants that cause hereditary breast cancer, ovarian cancer, or Lynch syndrome; and that 7 newborns had pharmacogenomics-associated variants [4]. Cancer is caused by the mutation of oncogenes and tumor suppressor genes under the influence of multiple genetic and environmental factors. Identification of key genes involved in tumor development and metastasis by high-throughput functional screening technologies will help to develop cancer treatment strategies. As a result, many technologies have been developed for the treatment of tumors at the genetic level [5,6]. Gene editing technologies aim to specifically change the target sequences of genetic material. It includes zinc-finger nuclease (ZFN), transcription activator-like effector nuclease (TALEN), and clustered regularly interspaced short palindromic repeat-associated protein (CRISPR/Cas) [7–9]. The progress of gene editing technology not only provides technical support for genetic research but also provides new therapeutic options for medicine.

The applications of ZFN and TALEN technologies in gene editing are limited by the complexity of vector assembly [10]. ZFN specifically binds to target genes by designing zinc finger domains and different ligation sequences of multiple zinc finger proteins (ZFPs), and then uses the restriction endonuclease Fok-1 for site-directed cleavage. For each new target sequence, specific tandem combinations of ZFPs need to be designed. A ZFP can only recognize specific triplet bases, which greatly increases the difficulty of designing specific ZFPs for target sequences. Additional extensive experimental validation is needed to ensure the specificity and efficiency of ZFPs. The whole assembly process is complex and inefficient. Based on the ZFN technique, a TALEN that can directly bind to DNA was fused to the DNA cleavage domain of Fok I restriction enzyme to construct a new generation of gene editing technology, TALENs. Unlike ZFNs, DNA recognition by individual TALEN motifs is largely independent of neighboring motifs, but because it recognizes a single base, the gene encoding TALENs is about 3 times longer than ZFNs. The large size of TALENs makes its packaging difficult. Alternatively, the invariance and high repetition of the TALEN consensus sequence results in inefficient overall assembly [11–13]. CRISPR/Cas is a third-generation gene editing technology [14]. CRISPR/Cas is an adaptive immune system that allows bacteria to efficiently recognize and cleave exogenous nucleic acids [15,16]. The CRISPR/Cas system has a variety of categories, among which the Type II CRISPR-associated nuclease 9 (CRISPR/Cas9) system is the most intensively researched and technically mature class. Owing to the ease of construction and precision of the CRISPR/Cas9 system, it has been applied in tumor treatment research [17–20]. Although CRISPR/Cas9 gene editing technology has made rapid progress, it has not yet been fully applied in clinical due to off-target effects and the lack of an effective delivery system [21,22]. A series of techniques have been developed to control the activity of the CRISPR/Cas9 system with spatiotemporal control to reduce off-target effects and toxicity [23].

Through continuous exploration of related research, a series of methods has been developed to deliver CRISPR/Cas9. The main methods include physical vector, viral vector, and non-viral vector delivery. Compared to physical and viral vector delivery, non-viral vectors have lower immunogenicity. Moreover, non-viral vectors are less payload-restricted and are often easier to synthesize [24]. Biomaterials are popular for the construction of non-viral vectors because of their versatility, biocompatibility, and high delivery efficiency [25]. With the development of nanotechnology, a variety of biomaterials have been developed as non-viral vectors [26]. To date, many excellent review articles have provided an overview of CRISPR/Cas9-specific vector applications [27–30]. However, to the best of our knowledge, reviews comparing CRISPR/Cas9 biomaterial delivery vectors and specifically introducing its application in tumor therapy are still lacking. In this context, we summarize the current status of the CRISPR/Cas9 system in the clinic and the trends of biomaterials as CRISPR/Cas9 delivery vectors for treating tumors. We also discuss the design, advantages, and disadvantages of these delivery methods to lay the foundation for the successful application of the CRISPR/Cas9 system in clinical tumor therapy.

## CRISPR/Cas9 Gene Editing System

### Introduction to the CRISPR/Cas9 gene editing system

The CRISPR/Cas system, derived from the genomes of bacteria and archaea, is a naturally acquired immune system that exerts immune defense primarily by recognizing the protospacer-adjacent motif (PAM) sequence [31]. CRISPR/Cas systems are divided into Class I and Class II based on the number of Cas proteins involved. Class I CRISPR/Cas system nucleases are composed of multiple subunits, and their major role is to recognize and cleave target nucleic acids using multi-Cas protein effector complexes [32]. Because the Class II system requires only one Cas protein to achieve efficient and specific cleavage of exogenous nucleic acids, it is the best choice for gene editing tools. The widely used Cas9 protein belongs to a Class II CRISPR/Cas system [33–36]. As early as 1987, Yoshizumi Ishino, a Japanese microbiologist, found abnormal repetitive sequences in the non-coding region of *Escherichia coli* [37]. A few years later, Mojica found a similar repeat in a halophilic archaebacterium and, together with Jansen, coined the name CRISPR [38]. In 2007, Barrangou et al. [39] demonstrated that CRISPR/Cas is a bacteria-acquired immune system through which bacteria build their own immunity by specifically recognizing invading foreign DNA and using Cas proteins to cut it. CRISPR/Cas9 was first successfully reconstructed in vitro in 2012, and the gene editing function was demonstrated in human cells in 2013, marking the beginning of the third generation of gene editing technologies [40]. Subsequently, the CRISPR/Cas9 gene editing technologies has undergone rapid development.

**Fig. 1.**
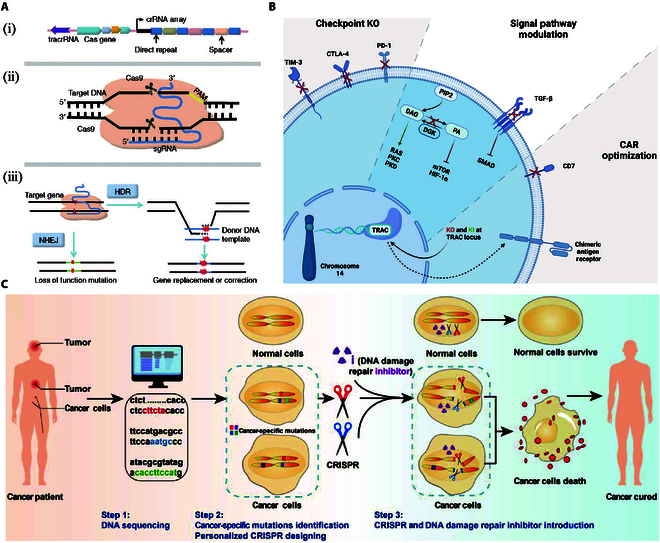
Structure of CRISPR/Cas9 and its application in tumor therapy. (A) Structure of CRISPR/Cas9. (i) Schematic of CRISPR locus (from *Streptococcus pyogenes*); (ii) role of the major domain of CRISPR/Cas9; (iii) HR and NHEJ repairing mechanism after double-strand break introduced by Cas9 [45]. Copyright 2016, Wei-Jing Dai et al. (B) CRISPR/Cas9 gene editing in T cells. The CRISPR/Cas9 system can be used to knock out immune checkpoint-related genes such as TIM3, CTLA-4, and PD-1, and can inhibit the immunosuppressive TGF-β signaling. CAR-T cells can be directly improved [65]. Copyright 2022, Elmas et al. (C) Kills cancer cells by inducing DNA double-strand break at the corresponding mutation sites [66]. Copyright 2022, Junfeng Jiang et al.

CRISPR/Cas9 comprises the nuclease Cas9 and guide RNA (sgRNA), in which Cas9 cuts DNA double strands while the sgRNA plays a guiding role [41]. Cas9 protein has 2 nuclease structural domains. The structure of Cas9 protein is mainly divided into the recognition lobe (REC lobe) and the nuclease lobe (NUC lobe). The REC lobe and NUC lobe are connected by the arginine-rich BH (bridge helix) domain. The REC lobe is composed of REC1, REC2, and REC3 and functions to connect other domains. The Bridge Helix domain is responsible for initiating shear. The NUC lobe consists of 3 domains, namely, RuvC domain, HNH domain, and PI domain. RuvC cleaves the target strand. HNH cleaves non-target strands complementary to sgRNA pairs. The PI domain is mainly related to the recognition of PAM [42]. The sgRNA is derived from CRISPR RNA (crRNA) and transactivated crRNA (tracrRNA) [43]. Each crRNA contains a conserved repetitive sequence complementary to the tracrRNA and a 20-nt transcribed spacer region complementary to exogenous DNA. The tracrRNA binds the Cas9 protein after complementation with crRNA, forming a CRISPR/Cas9–sgRNA complex that produces a double-stranded break at the target site in the genome (Fig. 1A) [44–46]. The CRISPR/Cas9 system specifically recognizes and shears foreign DNA or RNA in 3 stages: adaptation, expression, and interference [47]. When exogenous DNA, such as phage or plasmid DNA, enters bacteria, the Cas1–Cas2 complex recognizes the PAM sequence and specifically cleaves its adjacent target gene. Then, a new prototype spacer sequence is introduced into the CRISPR locus in the adaptation stage [48]. The expression stage is completed by the ribonuclease RNase III and Cas9 protein. RNase III processes the pre-crRNA (spacer sequences transcribe into precursor transcript RNA) into mature crRNA, which constitutes the sgRNA. Cas9 and the sgRNA together form the Cas9 ribonucleoprotein (RNP). At the end of the expression phase, each crRNA contains a spacer sequence that matches the original spacer sequence of the exogenous DNA [49,50]. During the interference phase, the exogenous DNA re-enters the cell and the PAM sequence of the exogenous DNA is recognized in 2 ways: (a) the sgRNA in the RNP recognizes a fragment complementary to the PAM sequence of the target DNA and (b) the Cas9 protein recognizes the PAM sequence on the exogenous DNA [51]. Finally, the RNP shears the target gene through the HNH and RuvC domains of the Cas9 protein. Broken double-stranded DNA can repair itself using 2 mechanisms: non-homologous end-joining (NHEJ) and homology-directed repair (HDR). In general, cells employ an efficient NHEJ to ligate broken DNA duplexes; however, repair is stochastic in nature, and the process usually involves mismatches of base insertions or deletions, resulting in frameshift mutations. If donor DNA with the same ends as the break sequence is provided as a repair template, the broken part of the genome will be repaired by HDR, resulting in the insertion of the repair template sequence [52,53]. CRISPR/Cas9 gene editing technology takes advantage of this process by artificially designing genomic sequences and guiding Cas9 nuclease to effectively cut DNA at specific locations. Repair after damage can result in gene knockouts or knock-ins to achieve genome modification.

### Applications of CRISPR/Cas9 in tumor therapy

#### Advantages of CRISPR/Cas9 in tumor

Compared with ZFN or TALEN, CRISPR/Cas9 has higher efficiency, lower construction cost, and wider research scope [54]. In CRISPR/Cas9, only the first 20 bases of the sgRNA are adjusted to identify different positions, reducing the construction cost required to modify the genome. In the CRISPR/Cas9 system, Cas9 and the sgRNA can function independently of each other. When Cas9 protein and multiple sgRNAs are expressed in cells, multiple genetic sites can be edited simultaneously, markedly increasing the editing efficiency. In addition, Cas9 functions as a monomeric protein, avoiding complex assembly [7]. The CRISPR/Cas9 gene editing technology has been applied in tumor to establish tumor models, explore drug targets, screen tumor metastasis-related genes, and develop treatments for tumor [55–57]. For example, the combination of CRISPR/Cas9 with single-cell multi-omics sequencing technology can be used to study tumor lineages and developmental microenvironments. CRISPR/Cas9 combined with gene mapping technology can be used for individualized diagnosis and treatment of patients with tumors [58]. Various gene mutations during cell development have been induced using CRISPR/Cas9 technology, and corresponding animal models have been established [59–62]. Furthermore, CRISPR/Cas9 can facilitate cell lineage tracing and transcriptome analysis in animal models to rapidly identify the mechanisms underlying the pathogenesis of many diseases [63,64].

#### Current research on CRISPR/Cas9 in tumor therapy

Tumor development and progression involve mutations and dysregulation of a series of genes, including oncogenes, tumor suppressor, and chemoresistance genes. The goal of tumor therapy is to restore the expression of mutational and dysregulated genes. There are 2 main strategies in which CRISPR/Cas9 can be applied in tumor therapy: (a) directly attack cancer cells by editing key functional genes or (b) indirectly attack cancer cells by enhance the viability of immune cells (Fig. 1B and C) [65,66].

Direct gene editing of tumor cells using CRISPR/Cas9 can involve the enhancement of tumor suppressor CRISPR/Cas9 to inhibit tumor progression and the targeted knockout of oncogenes to treat tumors [67]. Liu et al. [68] showed that targeted activation of the tumor suppressor genes using CRISPR/Cas9 led to bladder cancer cell apoptosis. The RNP, which can specifically recognize the viral genome, is used to directly target oncogenic viral genes. For example, knockdown of Epstein–Barr virus using CRISPR/Cas9 can significantly inhibit Burkitt lymphoma cell proliferation [69].

The CRISPR/Cas9 system can improve the immunotherapeutic susceptibility of tumors through multigene-targeting modification of T cells. Programmed cell death protein 1 (PD-1), cytotoxic T lymphocyte-associated antigen-4, T cell immunoglobulin, mucin domains-containing protein 3, and other common immune checkpoint genes affect T cell activation and facilitate tumor cell immune escape [70]. CRISPR/Cas9 can improve tumor immunotherapy by knocking out such immune checkpoint genes. In a study by Dong et al., the immune checkpoint gene, DEAH-box RNA helicase (DHX37), was found to inhibit T cell activation, cytokine production, and cytotoxicity in a mouse model of triple-negative breast cancer (TNBC) using CRISPR screening. In CD8-positive T cells, knockdown of DHX37 can improve adoptive immunotherapy in TNBC [71].

The CRISPR/Cas9 system can also play a therapeutic role by modifying chimeric antigen receptor T cells (CAR-T) [72]. CAR-T cell therapy has shown unprecedented efficacy in hematological malignancies [73]. However, the high complexity and cost of genetically engineered modified T cell technology and associated immune rejection have limited the applied of CAR-T cell therapy [74]. CRISPR/Cas9 can effectively knock out multiple genetic factors to produce generic T cells and improve the efficiency of CAR-T cell preparation. Generic CAR-T cells exhibit low graft rejection and improved biosafety [75]. Ren et al. used CRISPR/Cas9 to generate CAR-T cells lacking endogenous TCR, HLAI, and PD-1. Such CAR-T cells have reduced alloreactivity due to the deletion of TCR and HLAI class molecules and show potent antitumor activity [76]. In addition, CRISPR/Cas9 can enhance the function of CAR-T cells by knocking down genes encoding signaling molecules or T cell inhibitory receptors. In recent years, immunotherapy has shown great potential in tumor treatment. The combination of the CRISPR/Cas9 system and immunotherapy may further broaden the application of immunotherapy in patients with cancer. The CRISPR/Cas9 system provides a solid foundation for effective and innovative tumor research and therapy.

#### The challenge of CRISPR/Cas9 in tumor therapy

Although the CRISPR/Cas9 system has shown broad application prospects in the field of tumor therapy, challenges remain [77]. For instance, combinations of sgRNA and DNA sequences may result in the cleavage of DNA sequences other than the target sequence by the CRISPR/Cas9 system, resulting in harmful mutations. Studies have shown that cleavage can occur even with a 5-base mismatch between the sgRNA and DNA sequences, making the CRISPR/Cas9 system prone to off-target effects. Off-target effects can lead to pseudo-phenotypes, and unexpected editing may cause unwanted mutations and potential toxicity [78,79]. Detection methods for off-target effects include genome-wide double-stranded break detection and digested genome sequencing, which is a method for whole-genome sequencing of genomic DNA isolated by Cas9 digestion in vitro [80]. Although both methods have good sensitivity, they both face limitations. For example, genome-wide detection of DNA double-strand breaks is inefficient, while digenome-Seq is expensive and sequencing depth is limited when detecting multiple sgRNAs [81]. Thus, off-target effects remain an important issue for the future development of the CRISPR/Cas9 system.

A safe and effective delivery of CRISPR/Cas9 to the target cells is necessary. The delivery of the CRISPR/Cas9 system is often achieved by delivering the DNA sequence of the coding gene in the form of a plasmid or using a recombinant virus as a vector [82]. The introduction of exogenous DNA can easily cause permanent recombination in the genome, resulting in potential damage to endogenous genes and exacerbating the safety risks for clinical. The continuous expression of the CRISPR/Cas9 may lead to off-target effects and mutations [83]. The best way to deliver the CRISPR/Cas9 system is to directly deliver Cas9 and sgRNA. This delivery method can effectively reduce off-target effects [84,85]. However, Cas9 and sgRNA both exhibit poor stability in cells, and effective delivery to the nucleus is an urgent problem to be solved.

### Delivery strategies for the CRISPR/Cas9 system

There are 3 main delivery forms for the CRISPR/Cas9 system: (a) delivery of plasmid DNA encoding Cas9 and sgRNA, (b) delivery of Cas9 mRNA and sgRNA, and (c) delivery of Cas9 protein and sgRNA [86,87].

#### Delivery of plasmid DNA encoding Cas9 and sgRNA

The plasmid DNA (pDNA) delivering Cas9 and sgRNA can continuously and stably express Cas9 protein, sgRNA, and even the HDR repair template in cells. Moreover, double-stranded DNA is more stable and easier to manipulate than Cas9 protein and mRNA, making it a commonly used form of delivery [88]. Many physical methods, such as electric shock and injection, are applicable in this form. However, the large size and strong negative charge of pDNA cause great difficulties in loading and embedding [89]. pDNA must enter the nucleus for transcription, and the transcribed Cas9 mRNA must be transferred to the cytoplasm for translation and assembled with sgRNA before re-entering the nucleus to exert its function; this is a lengthy process that takes a long time to complete. In addition, for non-dividing cells, the plasmid requires a nuclear localization sequence (NLS) or cell-penetrating peptides (CPPs) to facilitate translocation into the nucleus [90].

#### Delivery of Cas9 mRNA and sgRNA

Rather than delivering Cas9 pDNA, Cas9 mRNA can be obtained using in vitro transcription and then transferred into the cell together with sgRNA. After delivering Cas9 mRNA and sgRNA to the target cells, Cas9 mRNA is translated into Cas9 protein and then combines with the sgRNA to form the RNP and accomplish gene editing. During gene editing, expression of the Cas9 protein is transient, resulting in lower off-target effects and faster action than the plasmid [91]. Currently, the main vectors for Cas9 mRNA delivery are cationic liposomes. The problem with this delivery strategy is that Cas9 mRNA is extremely unstable. In addition, RNA synthesized in vitro inevitably induces immune responses in vivo, resulting in the activation of pattern recognition receptors [92]. Suitable chemical modification of mRNA can not only prevent its degradation but also prevent an immune response. Yin et al. prepared e-sgRNA by modifying sgRNA with a 2' hydroxyl group and a thiophosphate ester bond. Compared to unmodified sgRNA, e-sgRNA exhibited higher editing efficiency when targeting Pesk9, Fah, and other genes in the mouse liver [93].

#### Delivery of Cas9 protein and sgRNA

The most efficient strategy is to deliver Cas9 protein and sgRNA directly. This delivery method can rapidly realize gene editing without transcription and translation and has better application prospects. RNP degrades rapidly and functions transiently that can reduce off-target effects and immune responses. It is difficult to deliver RNP because Cas9 has a large molecular mass and a positive charge. Additionally, obtaining large amounts of highly active Cas9 is challenging owing to the cost and bacterial endotoxin contamination [94,95]. When RNP is present on the target cell surface, anti-Cas9 T cells can attack the target cell [96,97]. Therefore, the design and preparation of the delivery system must focus on protecting the RNP from recognition by antibodies. Modifications of CPPs or NLSs in the delivery system also facilitate RNP entry into the nucleus for functional purposes.

### Delivery vectors for the CRISPR/Cas9 system

**Fig. 2.**
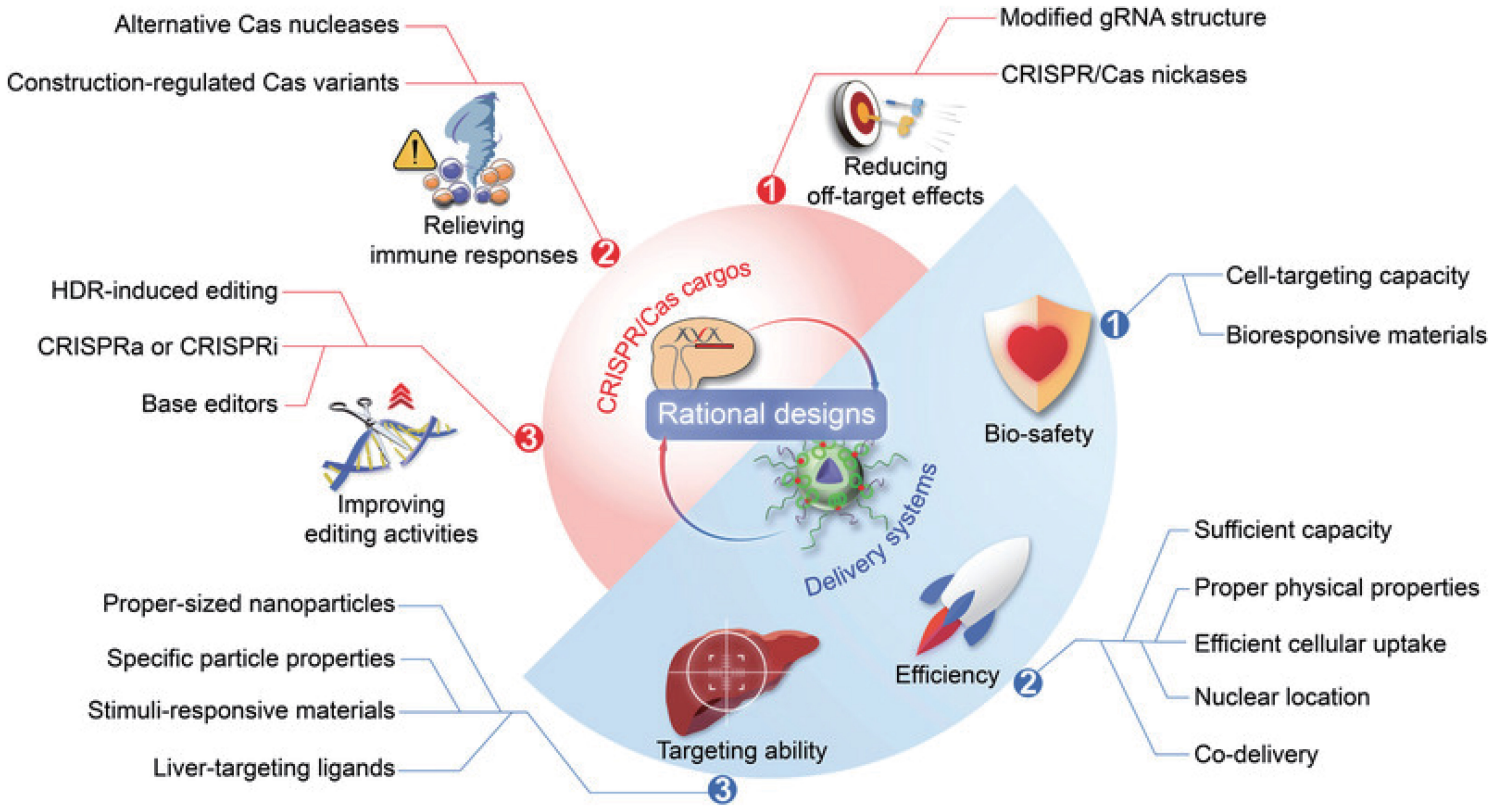
Design strategies for CRISPR/Cas9 biomaterial delivery systems [107]. Copyright 2021, Huimin Kong et al.

Efficient delivery of CRISPR/Cas9 directly affects gene editing efficiency. Delivery vectors for CRISPR/Cas9 systems can be divided into physical, viral, and non-viral vectors (Fig. 2A) [98,99].

#### Physical delivery of the CRISPR/Cas9 system

Physical delivery needs equipment to directly deliver the CRISPR/Cas9 complex to the cytoplasm or nucleus, which can rapidly cleave the targeted DNA and degrade quickly. Physical delivery methods include electroporation, hydrodynamic tail vein injection, microinjection, ultrasonic microbubbles, and lasers. Physical delivery can be applied to a wide range of cells. For example, only electroporation was reported to be successful when CRISPR/Cas9 was delivered to refractory primary T cells [100]. Schumann et al. [101] successfully achieved nearly 40% gene editing efficiency and up to 20% gene knock-in efficiency by delivering CRISPR/Cas9 to primary T cells via electroporation. However, these physical approaches are usually impractical for in vivo therapy because of their high cytotoxicity and the need for specific equipment. For example, electroporation, which is commonly used as a physical delivery method, is often only suitable for in vitro cell transfection. Although microinjection can be used for cell transfection in vivo, it is harmful to cells and has low throughput; therefore, it is often only suitable for the transfection of embryonic cells. In short, although physical delivery methods are simple and efficient, they are only suitable for in vitro experiments. Its toxicity limits its usefulness as an efficient delivery system in vivo.

#### Viral delivery of the CRISPR/Cas9 system

Viral vectors involve packaging DNA, encoding the system components into a virus, and delivering it to cells. It includes adenoviruses (AdVs), adeno-associated viruses (AAVs), lentiviral vectors (LVs), and bacteriophages. AdV infects cells and elicits a strong immune response in animals. AAVs exhibit lower immunogenicity than other viral vectors [102]. Bengtsson et al. [103] demonstrated that AAV delivered the CRISPR/Cas9 targeting muscle-related genes and achieved HDR-mediated gene editing in a Duchenne muscular dystrophy model. When using LVs as the delivery vector for CRISPR/Cas9, host genome integration may lead to unnecessary off-target insertion mutations, which pose safety risks. Most laboratories currently do not have the ability to prepare non-genomic integrated LV vectors; therefore, LVs are most commonly used to construct disease models due to laboratories to prepare with difficulty. Bacteriophage applications are relatively rare owing to their narrow host selectivity. Overall, viral vectors have high transfection efficiency, but their high mutation rate and carcinogenic risk make their clinical application challenging. In addition, the limited viral loading capacity makes it difficult for efficient delivery. The emergence of engineered virus-like particles is expected to improve the delivery efficiency of viral vectors to the CRISPR/Cas9 system [104].

#### Non-viral delivery of the CRISPR/Cas9 system

Non-viral vectors are constructed using synthetic vector materials to mediate gene transfer. The non-viral vectors not only have high packing capacity and easy assembly but also impose fewer restrictions on the payload and are generally easier to synthesize [24]. Many non-viral vectors are based on hybrids of different types of materials; combining the advantages of various materials allows for improved delivery efficiency, and several advances have been made in in vivo and in vitro studies [105,106]. Biomaterials have gained popularity as non-viral vectors owing to their versatility, biocompatibility, and increased transfection efficiency. The rapid development of nanomaterials has shown great promise for the delivery of the CRISPR/Cas9 system in clinical settings (Table).

**Table.** Biomaterial vectors for delivery of the CRISPR/Cas9 system

Methods	Characteristic	Advantages	Disadvantages
pH-responsive liposomes [115,116]	Form pH-responsive controlled-release systems	Simple preparation; high stability; targeted drug delivery	Easily degraded
Thermosensitive liposomes [117,118]	Form temperature-responsive controlled-release systems	Targeted delivery; cause local high temperature to directly kill tumor cells	Easily degraded; high heat damages normal tissues
Light-responsive liposomes [119]	Form light-responsive controlled-release systems	Convenience; targeted delivery; photodynamic therapy; photothermal therapy; imaging	Easily degraded; low efficiency
Cationic liposomes [120–122]	Neutralize the negative charge carried by the mRNA and assist the mRNA molecules to cross the cell membrane	Targeted delivery; long-acting; low toxicity	Easily degraded; instability
Ionizable lipids [123,124]	The polarity changes with pH	Low toxicity; low immunogenicity	Instability; low efficiency
Polyethyleneimine [129–131]	A high density of positive charges	High transfection capacities	Toxicity
Nanocapsules [132–134]	Released by changing its porosity or breaking down its structure in response to environmental stimuli	Convenient purification, storage and transportation; convenient dose control	Low efficiency; toxicity
Chitosan [135–139]	Biodegradable natural polysaccharide	Low toxicity; good biocompatibility and biodegradability	Low efficiency; instability
Dendrimers [140,141]	Highly branched molecules with 3-dimensional structures	Low dispersion; high performance; easy to modify	Toxicity; high cost
Gold nanoparticles [147–149]	Adjustable size and shape; optical reactivity; easy surface modification	High targeting; high binding; long circulation half-life; good biocompatibility; fast tumor cell uptake	Toxicity; high cost
Zeolitic imidazole frameworks [151–153]	The coordination of transition metal ions and imidazole to form a zeolite topology	Easy modification and functionalization; pH sensitivity; high biocompatibility	Aggregation in body fluids; toxicity; low efficiency
Graphene oxide [155]	Near-infrared photothermal conversion ability	Good biocompatibility; good stability	Toxicity
Black phosphorus [156]	Multiple fold structure; large specific surface area	Used in photothermal therapy and photodynamic therapy	Instability; easily oxidized
Mesoporous silica nanoparticles [157,158]	Large surface area; regular hole; easy surface modification	High load capacity; controlled-release rate; flexible trigger release platform; high biocompatibility and stability	Toxicity; the generation of reactive oxygen species increased; difficult to degrade
Nanodiamond [159–163]	With adsorbent properties	Good biocompatibility; low toxicity	High surface energy; easy to agglomeration
DNA nanowires [164,165]	Yarn-like nanoparticles by rolling cycle amplification	High efficiency, low toxicity, good biocompatibility	Complex preparation; high cost
Nanogels [166,167]	The 3-dimensional network nanostructure	Avoiding premature release; low accumulation	Complex preparation; immunogenic
Exosomes [168,169]	A natural mode of intercellular communication	Good biocompatibility	Complex preparation

Importantly, the effective application of CRISPR/Cas9 requires careful design of delivery vectors and selection of appropriate biomaterials. In general, the choice of delivery vector strategy should consider 3 aspects: biosafety, delivery efficiency, and targeting ability (Fig. 2B) [107]. For biosafety, the choice of vector should be biocompatible and minimize immune harm. Besides, the carrier should have cell targeting ability to minimize the impact on other non-disease sites and reduce potential harm [108]. Improving the delivery efficiency of vectors can be considered from the following aspects. The vector should have sufficient capacity to protect CRISPR/Cas9 from stable encapsulation. Appropriate physical properties of the vector (e.g., size, potential) help improve delivery efficiency. In addition, select materials promote the release of intact CRISPR/Cas9, such as lipid-mediated membrane fusion, cationic polymer-mediated swelling, and membrane instability. Finally, combination delivery of multiple materials could synergistically improve efficiency. The vector should have an efficient targeting capability. Targeting can be improved by decorating the carrier with targeting ligands, or by using materials that respond to external specific signals [109,110]. The selection of appropriate materials to deliver the Cas9 system is not only the prerequisite of gene editing therapy, but also the guarantee of treatment efficiency.

## Biomaterial Vectors for CRISPR/Cas9 Delivery

### Liposomes/lipid nanoparticles

The liposome is mainly composed of phospholipids and cholesterol. When the phospholipid molecules are dispersed in water, the hydrophilic head and hydrophobic tail are close to each other, forming a closed vesicle [111]. Liposomes can deliver the CRISPR/Cas9 system across cell membranes through endocytosis or micropinocytosis while protecting the components from enzymatic degradation and immune responses. However, the complex preparation process, low efficiency, and poor stability of liposomes in vivo limit the development as delivery systems. With the development of nanotechnology, the evolution from liposomes to multifunctional lipid nanoparticles (LNPs) has begun.

**Fig. 3.**
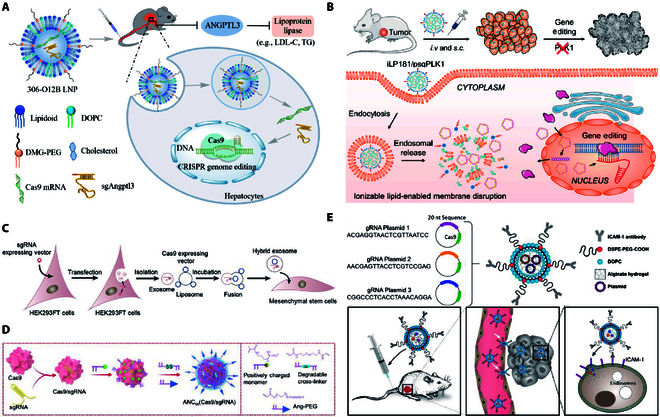
Biomaterial vectors for CRISPR/Cas9 delivery. (A) Design of the lipid nanoparticle-based CRISPR/Cas9 delivery system [112]. Copyright 2021, Min Qiu et al. (B) Ionized lipids deliver the CRISPR/Cas9 system [123]. Copyright 2021 Chunhui Li et al. (C) Exosome–liposome hybrid nanoparticles deliver the CRISPR/Cas9 system [125]. Copyright 2018, Yao Lin et al. (D) Nanocapsules for delivery of Cas9 ribonucleoprotein complexed with sgRNA [133]. Copyright 2018, Yan Zou et al. (E) The structure of a noncationic and deformable nanolipogel and its in vivo delivery mechanism of CRISPR/Cas9 for gene editing [167]. Copyright 2019, Peng Guo et al.

LNPs are a kind of nanoparticle carrier for drug delivery to cells, which is composed of ionizable lipids, neutral auxiliary phospholipids, cholesterol, and polyethylene glycol (PEG) lipids (Fig. 3A). LNPs are a pH-dependent cationalized lipid that better overcomes their systemic toxicity. The PEG lipids improve particle stability and shield immune recognition. The auxiliary phospholipids can stabilize the structure and cholesterol can promote fusion with the cell membrane. LNPs are considered to be less immunogenic [112]. The advantages of good DNA affinity, stability in the blood, and strong membrane fusion ability render LNPs the most promising CRISPR/Cas9 delivery system. Indeed, the efficiency of Cas protein and sgRNA delivered by LNPs in human cells has been reported to reach 80% [113]. With the development of a variety of LNPs, liposome technology has ushered in a new breakthrough. A series of stimulus-responsive liposomes have emerged. In addition, more efficient vectors can be designed by combining liposomes with other biomaterials to exert their respective advantages.

#### Stimuli-responsive liposomes

In recent years, many researchers have investigated responsive liposomes, which are liposomes that undergo structural changes when exposed to microenvironmental stimuli in vivo, such as pH, light, temperature, magnetic field, or specific enzymes. Such liposomes allow CRISPR/Cas9 to be released under specific conditions, thereby improving gene editing efficiency [114].

In the tumor microenvironment, the pH is 5.8 to 7.2 outside the cells, and about 5.5 in the lysosomes or endosomes, both of which are more acidic than normal tissues. Therefore, pH differences can cause carrier chemical bond breaking, charge changing, conformational changes, etc. Based on this basic theory, pH-responsive liposomes were constructed. For example, Zhen et al. constructed pH-responsive liposomes by introducing pH-responsive peptides or polymers into traditional liposomes, and the pH-responsive characteristics of the lipids allowed for the specific release of the CRISPR/Cas9 system at the tumor site. Conventional drug delivery liposomes do not specifically recognize this pH change when they enter circulation, resulting in their indiscriminate effects on cells. pH-responsive liposomes maintain a stable structure in a neutral environment and can effectively encapsulate drugs or genes, which are then released following conformational changes induced by an acidic environment [115]. Hu et al. constructed a hybrid nanoparticle using a pH-responsive lipid and poly acid. The pH-responsive liposomes were not only rapidly taken up by dendritic cells but also rapidly disintegrated and released antigens in a buffer similar to that within dendritic cell endosomes [116]. The pH-responsive liposomes can be targeted to the acidic tumor environment and degrade the original nanoparticles. The specific activation of nanomedicine in the tumor microenvironment is achieved for the selective killing of tumor cells.

Thermosensitive liposomes (TSLs) are often used as chemotherapeutic drug carriers that can trigger the release of drugs [117]. TSLs are often composed of dipalmitoyl phosphatidylcholine and/or distearoyl phosphocholine in different ratios. Terence et al. designed a novel copolymer TSL using the thermosensitive polymorph and the pH-responsive polymorph propylacrylic acid, which both reduced the required therapeutic dose of heat and collateral damage and provided pH sensitivity to the acidic tumor microenvironment. This work improved the precision delivery of chemotherapy drugs and expanded the application prospects of polymer-designed TSLs [118]. TSLs can not only realize the selective release of temperature sensitivity to achieve the purpose of targeted drug delivery, but also cause local high temperature to directly kill tumor cells. It should be noted that excessive heating time may also cause damage to normal tissues. In the current study, the TSLs combine with other materials to form multiple responsive liposomes to realize tumor diagnosis and treatment, for example, photothermal responsive liposomes, thermal responsive magnetic liposomes, pH temperature-sensitive liposomes, etc.

External light is a convenient stimulus. External light is easily regulated in terms of light timing and light location, which provides additional benefits for precisely adjusting cargo release. Hamada et al. investigated a light-responsive lipid for plasmid delivery. In this study, they condensed Cas9/sgRNA plasmids with TAT peptide-bound gold nanoparticles (AuNPs) and then coated the complex core with a lipid bilayer consisting of 1,2-dioleoyl-3-trimethylammonium-propane, dioleoylphosphatidylethanolamine, cholesterol, and distearoyl phosphoethanolamine (DSPE)-PEG2000. Due to the photothermal characteristics of AuNPs, the integrity of the lipid membrane can be destroyed by 514-nm laser irradiation, and the effective release rate can reach 79.4%. In addition, they injected light-responsive LNPs into mouse tumors and found that tumor growth was significantly reduced after light irradiation [119]. Light-responsive liposomes can be applied to photodynamic therapy, photothermal therapy, and photoactivated drug release of tumors by taking advantage of their optical properties, which is conducive to the diversification and specific treatment of tumors. In addition, light-responsive liposomes can also be used for photoacoustic imaging and photothermal imaging to improve the accuracy of early diagnosis of tumors.

#### Cationic liposomes

The lipid molecules originally used to construct liposomes are cationic lipids. The head group of the cationic liposomes carries a permanent positive charge, which can neutralize the negative charge carried by the mRNA and assist the mRNA molecules to cross the cell membrane. Cationic lipid-based nanoparticles are commonly used to deliver CRISPR/Cas9 systems. Jiang et al. established a novel cationic LNP system that effectively delivered CRISPR/Cas9 to the liver, cut the covalently closed loop DNA in the hepatitis B virus (HBV) genome and the endogenous target Pcsk9 gene in cells, and reduced the expression of HBV-related antigens [120]. Zhang et al. [121] synthesized a PEGylated phospholipid-modified cationic LNP delivery system that effectively concentrated and encapsulated pDNA in a core–shell structure, which achieved a 47.4% transfection efficiency in vitro and inhibited the growth melanoma cells in mice. Liu et al. reported a bioreducible LNP (BAMEA-O16B) with a hydrophobic tail containing disulfide bonds. BAMEA-O16B can encapsulate CRISPR/Cas9 through electrostatic interactions, and the disulfide bond is reduced by glutathione in the cell, thereby promoting the release of Cas9 mRNA and sgRNA. Approximately 90% effective gene editing was achieved by CRISPR/Cas9 delivery via BAMEA-O16B [122]. Cationic liposomes as a CRISPR/Cas9 system delivery vector have the advantages of targeting, being long-acting, and having low toxicity. In tumor therapy, cationic liposomes are used to deliver mRNA, which can efficiently cross the cell membrane and nuclear membrane, transfect cells, and make them express/silence genes. At present, many studies have been conducted to improve the stability, targeting and transfection efficiency of cationic liposomes by structural modification of the surface, and the use of new preparation processes.

#### Ionizable lipids

The emergence of ionizable lipids is an important breakthrough in the development of LNP. The polarity of ionizable lipids changes with pH. Huang et al. developed a novel ionizable LNP (iLP181) for delivery of Cas9 and sgRNA plasmids. iLP181 consists of the lipid iLY1809, cholesterol, phospholipids, and PEG lipids. They demonstrated that iLP181 could package CRISPR/Cas9 to form a stable and homogeneous iLP181/psgPLK1 nanodrug system (Fig. 3B) [123]. Professor Dan encapsulated Cas9 mRNA and GFP sgRNA with a novel ionizable amino lipid. The encapsulation efficiency of the resulting sgGFP-cLNPs can reach more than 90%. The gene editing reaches up to 98% in vitro in a variety of cancer cells, which can effectively inhibit tumor growth and improve survival [124]. Although ionizable lipids have lower cytotoxicity and higher gene silencing efficiency than other liposomes, they are not stable for a long time because their charge is affected by the pH change in the environment. The pH of blood is 7.35 to 7.45; thus, the ionizable liposomes are unstable during the process of mRNA delivery, which affects the final delivery efficiency.

#### Lipid combined with other materials

The combination of lipid and other materials, such as protamine, exosome, polymers, and AuNPs, can be used to design efficient carriers. Zhang et al. combined pCas9/sgRNA with protamine and chondroitin sulfate, and the outer-coated cationic liposomes protected the plasmid from nucleic acid degradation. Modification of DSPE-PEG on the surface of the LNP using the back-insertion method produced PEG-LNP (PLNP). Intratumoral injection of PLNPs carrying pCas9 and an sgRNA targeting POLL-like kinase-1 (PLK-1) reduced PLK-1 levels in A375 cells and inhibited tumor growth by 67% [121]. Lin et al. hybridized liposomes with exosomes and obtained hybrid nanoparticles with high encapsulation efficiency. The hybrid nanoparticles express genes that could not be transfected with liposomes alone in mesenchymal stem cells (Fig. 3C) [125].

### Polymers

Polymers can be used to encapsulate genes and drugs via electrostatic attraction and recombination with negatively charged nucleic acids. Polymeric carriers have been suggested to enter cells via the clathrin-mediated endocytosis mechanism. Polymeric carriers are easy to synthesize, safe, non-immunogenic, and widely used in delivery [126,127].

#### Polyethylenimine

Polyethylenimine (PEI) is a cationic polymer with a high density of positive charges due to its -NH- group, which can bind to negatively charged nucleic acids through electrostatic interactions and mediate efficient transfection. Branched and linear forms of PEI exhibit high transfection capacities both in vivo and in vitro [128]. Branched PEI deliver plasmids that targeting Slc26a4 was successfully and edited at approximately 22.9% efficiency [129]. Limitations regarding further application of PEI lie in its cytotoxicity; combining PEI with biodegradable molecules, such as heparin, polycaprolactone, dextran, chitosan, or folic acid, is a potential strategy for reducing its toxicity [130]. Zhang et al. [131] explored polyethylenimine-β-cyclodextrin (PC) as a vector for plasmid delivery. The PC could form nanocomplexes with plasmids, which enabled efficient gene editing in HeLa cells. PEI has a high positive charge property, which can adsorb and compress negatively charged genes into positively charged gene/vector complexes on the surface, and aggregate in the tumor tissue area through the enhanced permeability and retention (EPR) effect to achieve targeted delivery. However, PEI with high molecular weight is difficult to degrade after entering cells, and its long-term accumulation in vivo will produce potential cytotoxicity. The targeting, degradability, and biocompatibility of PEI can be effectively improved by modification.

#### Nanocapsules

Nanocapsules, the common form of polymer nanoparticles, refer to cavities surrounded by a polymer membrane or shell. The shell of the nanocapsule has many functions, such as protecting the core from environmental damage, increasing the stability of the nanocluster, increasing the activity of the substance, and changing the optical, electrical, and magnetic properties of the core. Chen et al. introduced cationic monomers, anionic monomers, imidazole-containing monomers, N'-bis(acryloyl)cystamine crosslinkers, and PEG coupled with ligands on the surface of the Cas9/sgRNA complex to prepare biodegradable, covalently crosslinked polymers coated with Cas9/sgRNA, called nanocapsules. Cationic and anionic monomer mixtures are adsorbed around Cas9/sgRNA via electrostatic interactions, and other molecules are attracted to the Cas9/sgRNA surface via hydrogen bonding and van der Waals forces [132]. Zou et al. [133] developed a novel nanoparticle that safely and effectively delivered CRISPR/Cas9 non-invasively to the brain and targeted glioma cells, achieving an editing efficiency of 38.1% and significantly prolonging the survival time of glioma mice (Fig. 3D). Meng et al. [134] reported a technique for preparing nitrogen-doped, carbon-coated cuprous oxide hollow nanocapsules (HCONCs), which exhibited excellent antitumor effects, indicating that HCONCs may be an effective anti-cancer strategy. Nanocapsules can release active compounds in response to environmental stimuli by changing their porosity or decomposing their structure. In addition, nanocapsules can be freeze-dried, so they can be easily purified, stored, and transported as powders, while providing flexibility for dose control.

#### Chitosan

Chitosan is an abundant, non-toxic, and biodegradable natural polysaccharide obtained via the N-deacetylation of chitin [135,136]. Chitosan has good biocompatibility but a relatively low transfection efficiency. Generally, the interaction between chitosan and the cell surface can be increased by combining chitosan with 5β-bile acid, deoxycholic acid, and stearic acid to improve its transfection efficiency [137,138]. Zhang et al. used lactate-targeted chitosan nanoparticles (CLPV NPs) to deliver sgVEGFR2/Cas9 plasmids and paclitaxel (PTX). CLPV NPs sustained release on HepG2 cells and had good cellular uptake. In HCC and HepG2 cells, co-delivery of the plasmid and PTX nanocomposites showed a synergistic therapeutic effect, and the genome editing efficiency reached 38%. CLPV NPs were found to cause greater than 70% inhibition of tumor progression in a mouse model of hepatoma-22 cells [139]. Although chitosan has great advantages as a Cas9/sgRNA delivery vector, it also has some limitations. For example, chitosan contains many hydroxyl, intermolecular, and intramolecular hydrogen bonding, resulting in the poor solubility of chitosan. Therefore, it limits its interaction with sgRNA to a certain extent.

#### Dendrimers

Dendrimers are highly branched molecules with 3-dimensional structures. Branching of the dendrimer and surface groups increases with the number of dendrimer generations. Dendrimers exhibit very low dispersibility and high performance. Owing to their structural advantages, dendrimers are used to construct different types of drug vectors and combine the characteristics of various treatment strategies to achieve efficient and precise treatments [140]. The anti-cancer drugs doxorubicin (DOX) and methotrexate (MTX) were packaged into polyamino-ester (PAE) dendrimers and polyamide (PAMAM) dendrimers. DOX-PAE/lipid complexes induce the dendritic molecules’ release. Changes in the microenvironment lead to MTX-PAMAM/lipids release. Both drugs at the tumor site enhance their anti-cancer synergy [141]. It is important to note that the synthesis of dendrimers is complex. In particular, high algebra dendrimer polymers have large steric hindrance. High production costs make it more difficult to industrialize. In addition, cation-induced toxicity also limits its medical applications.

### Inorganic nanoparticles

Inorganic nanoparticles are synthesized using inorganic particles and biodegradable polycations. Inorganic nanoparticles can encapsulate drugs or biomolecules, cross cell membranes via endocytosis, deliver drugs to target tissue, and release drugs to treat diseases [142]. Inorganic nanoparticles can also be used as gene vectors to encapsulate, concentrate, and protect nucleic acids from nuclease degradation. For example, the porous structure of mesoporous silica can encapsulate nucleic acids within particles, reducing the degradation of nucleic acids and improving the loading efficiency. Li et al. [143] loaded siRNA with magnetic mesoporous silica and modified PEI on its surface. This vector can effectively protect the siRNA and show efficient knockdown of the endogenous B cell lymphoma 2 gene. The efficiency of mesoporous silica to load nucleic acids is correlated with the large pore size. Kim et al. reported a very large pore size mesoporous silica (>15 nm). Compared with mesoporous silica with a smaller pore size (∼2 nm), mesoporous silica with a larger pore size can load nucleic acids more efficiently and effectively protect them from nuclease degradation [144]. Moreover, inorganic nanoparticles have a large specific surface area, good biocompatibility and degradability, easy preparation, convenient storage conditions, and good stability [145,146]. Therefore, inorganic nanoparticles have broad application prospects as delivery vectors for CRISPR/Cas9 applications.

#### Gold nanoparticles

AuNPs are an effective platform for drug delivery because their optical properties are dependent on the shape and size of the nanoparticles and their surface chemistry is easily modified [147]. AuNPs can be loaded with small-molecule drugs, peptides, proteins, interfering RNA, DNA, etc. Mout et al. used arginine-AuNPs to design Cas9 protein/sgRNA nanopolymers. The constructed delivery system was able fuse with target cells and release Cas9 protein and sgRNA directly into the cytoplasm. The delivery efficiency of this method was greater than 90% and the editing efficiency was as high as 30% [148]. Lee et al. used AuNPs to design a new vector, CRISPR-Gold, which can successfully deliver Cas9/sgRNA into refractory primary cells and stem cells. CRISPR-Gold also efficiently induced HDR in the muscle tissue of mice in vivo, correcting 5.4% of the dystrophy gene in mice, which was approximately 18 times more effective than direct treatment with Cas9/sgRNA and donor DNA [149]. Due to the unique optical properties and versatility of AuNPs, they exhibit high targeting, high binding, longer circulation half-life, good biocompatibility, and fast uptake by tumor cells. AuNPs are mainly used for the delivery of antitumor drugs, photoacoustic imaging, and photothermal imaging, and as a photothermal contrast agent for photothermal therapy to kill cells [150].

#### Metal-organic frameworks

Metal-organic frameworks (MOFs) are multifunctional hybrid materials comprising 3-dimensional crystalline networks of metal nodes and organic pillars. With micropores capable of accommodating guest molecules, MOFs are highly functional inorganic nanoparticles for molecular storage, separation, and catalysis [151]. Zeolitic imidazole frameworks (ZIFs), emerging organic–inorganic hybrid polymers, are a subtype of MOFs characterized by the coordination of transition metal ions and imidazole (or imidazole derivatives) to form a zeolite topology. ZIFs are characterized by easy modification and functionalization, pH sensitivity, and high biocompatibility and, thus, provide an excellent delivery platform for drugs, imaging probes, and biological macromolecules [152]. Yang et al. designed ZIF-90/Cas9 nanoparticles that formed via the imidazole-2-formaldehyde with Cas9. This delivery system achieved 35% gene editing efficiency in HeLa cell lines [153]. At present, there are various synthesis methods for ZIFs, and the structures and properties of ZIF prepared by different synthesis methods are not consistent. However, the size and surface properties of ZIFs are the main factors affecting their delivery efficiency. It is of great significance to explore the preparation method with good feasibility, high reproducibility, and high delivery efficiency for ZIFs drug delivery. In addition, ZIFs also face problems such as aggregation in body fluids and the effects of metal ions generated after metabolism on the human body.

#### Graphene oxide and black phosphorus

Graphene oxide (GO) is a stable biocompatible that controlled the release of functional proteins [154]. The condensing agent 1-(3-dimethylaminopropyl)-3-ethylcarbodiimidehydrochloride was coupled to PEG and PEI to form an amide to produce a GO-PEG-PEI vector. This vector achieved 39% gene editing efficiency in a human gastric adenocarcinoma cell line [155]. As a loading substrate, GO is less toxic, and its large specific surface area allows for high loading. PEG modification can further enhance the biocompatibility and stability of GO. After modification with positively charged PEI, GO-PEG-PEI can electrostatically bind to negatively charged sgRNA. The absorbance of GO extends from the ultraviolet region to the infrared region, which gives it excellent near-infrared photothermal conversion ability. The photothermal properties of GO can be used not only to construct nanoparticles that can respond to exogenous stimuli, but also to realize photothermal therapy of tumors. Therefore, GO nanotherapeutic platforms can be designed as stimulation-responsive smart drug delivery systems for targeted mRNA delivery and comprehensive tumor therapy. However, the biosafety of GO is still a huge challenge. At present, the biosafety assessment of GO is mainly in the in vitro experiment and animal experiment stage, and there is a lack of relevant clinical application data.

Black phosphorus (BP) is a thermodynamically stable form of phosphorus with a 2-dimensional folded honeycomb structure that provides a large specific surface area. BP provides an ideal anchoring site for Cas proteins, indicating its potential for loading and delivering biomolecules. The loading capacity of RNP on BP nanosheets was reported to be 98.7%, and effective tumor-related gene editing was achieved at a low nanoparticle concentration, reflecting a good loading capacity [156]. The excellent near-infrared optical properties of BP have been applied to photothermal therapy of tumors, and the synergistic effect of photothermal therapy and gene therapy has been realized. However, BP is chemically active and easily reacts with oxygen and water, resulting in rapid degradation, which has become one of the biggest obstacles to its application in biomedicine.

#### Mesoporous silica nanoparticles

Mesoporous silica nanoparticles (MSNs) are mesoporous materials with regular structures. MSNs are stable and biocompatible and can be degraded in vivo. The porous internal structure of MSNs endows them with good drug loading capacity. MSNs are easily prepared, modified, and encapsulated. The internal pore shape and size are controllable, and the particle size can be controlled [157]. Zhang et al. prepared nanoparticles coated with mesoporous silica. In a mouse model of hepatocellular carcinoma, they demonstrated that the vector did not damage organs, and EGFR editing efficiency was greater than 60%, which caused 85% tumor suppression [158]. At present, the main challenge of MSNs for CRISPR/Cas9 delivery is that its stable framework structure makes it difficult to degrade and to be excreted in the body, resulting in long-term accumulation in the body and can easily cause damage.

#### Nanodiamonds

Nanodiamonds (NDs) have adsorbent properties that can adsorb various substances on their surface, including DNA, proteins, small-molecule drugs, and chemicals. Based on this property, targeted drug delivery systems with long-term therapeutic effects can be developed. In addition, the biocompatibility and toxicity profiles of ND make it a fairly safe delivery vector [159–161]. The effectiveness of ND in the context of cancer has been demonstrated. For example, interference of the TGF signaling pathway using ND can reduce the invasiveness of tumor cells, prevent their acclimatization to macrophages, and inhibit tumor metastasis [162]. Furthermore, Yang et al. used ND as the CRISPR/Cas9 vector targeting the RS1 gene, mutations in which cause inherited retinal diseases. Two linear DNA constructs were designed: RS1-sgRNA and Cas9-GFP. The results demonstrated that RS1 gene editing and inherited retinal diseases occurred in cells [163]. ND has the following characteristics: large specific surface area, high surface energy, and easy agglomeration, which limit its application in tumor therapy. At present, ND is mostly functionalized to enhance its dispersion. The modified ND can be used not only as carriers for drug delivery, but also for fluorescent probes and biological imaging.

### DNA nanowires/nanogels

DNA nanowires are yarn-like DNA nanoparticles synthesized by rolling cycle amplification. Base pairing between DNA nanowires and Cas/sgRNA complexes via complementary sequences results in robust but reversible interactions that can destroy target genes while maintaining cell viability, thereby improving the efficiency of gene editing [164]. Sun et al. found that DNA nanowires have good biocompatibility and are less toxic to the human body than other drug delivery systems made of synthetic materials. In human osteosarcoma cells (U2OS), DNA nanoparticles can carry drugs and target tumor cells with a gene editing efficiency of 36%. Furthermore, in EGFP U2OS mice, DNA nanoparticle treatment could effectively achieve gene knockout [165]. Due to their structural programmability, good biocompatibility, and cell membrane permeability, DNA nanowires have become an excellent platform for studying drug delivery to cancer cells. However, the application of DNA nanowires to the clinic has been less studied and it is still in the preliminary stage.

Nanogels are intramolecularly crosslinked nanopolymer gels composed of hydrophilic or amphiphilic polymer chains. The 3-dimensional network nanostructure of nanogels exhibits high structural stability. Nanogels can withstand dissociation or aggregation caused by dilution and interactions with various components in the bloodstream after CRISPR/Cas9 administration, thereby reducing premature drug release and drug accumulation at the tumor site [166]. Guo et al. prepared an intercellular cell adhesion molecule-1 antibody-modified tumor-targeted nanolipogel system and demonstrated that systemic administration effectively knocked out lipocalin 2 in TNBC, with a tumor cell inhibition rate of 77% (Fig. 3E) [167]. In addition to good biocompatibility, biodegradability, and receptor-specific binding properties, nanogels can also improve the water solubility of hydrophobic drugs and prolong the circulation time of drugs in vivo. The properties of nanogels with different hardness are different. Using the mechanical properties of nanogels to overcome the removal of the reticuloendothelial system function, achieving mRNA maximum efficiency delivery is worth exploring.

### Exosomes

Exosomes are intercellular communication vehicles that are often used as delivery vectors for small molecules and proteins [168]. For example, tumor-derived exosomes can be loaded with CRISPR/Cas9 expression plasmids by electroporation in vitro and used as delivery vectors; however, their application is limited owing to their small size and relatively weak loading capacity. Lin et al. hybridized cationic liposomes (Lipofectamine 2000) with exosomes to form hybrid exosomes, which exhibited good chemotaxis and stability. These hybrid exosomes had strong liposome-loading capacity and were shown to efficiently deliver CRISPR/Cas9 system components to mesenchymal cells [125]. Usman et al. produced a large number of red blood cell (RBC)-derived extracellular vesicles (RBCEVs) and demonstrated that RBCEV-mediated RNA drug delivery resulted in efficient gene editing in cancer cells without any cytotoxicity. This finding may address the issue of the cellular source of exosomes as a delivery system for gene therapy [169]. Exosomes have natural advantages over other synthetic biomaterials for delivering the CRISPR/Cas9 system. First, exosomes are endogenous nanoparticles with low immunogenicity and thus high safety. Second, exosomes can be circulated to all compartments of the human body. For example, they can cross the blood–brain barrier to complete the delivery to the central nervous system. Finally, exosomes can be easily engineered and modified to better achieve the purpose of delivery. However, exosomes have low delivery efficiency as CRISPR/Cas9 delivery vectors. Thus, a large amount of exosomes is required to achieve a therapeutic dose. Improving the loading of active ingredients has become the primary and key challenge in the research of exosomal delivery.

## Applications of Biomaterial-Based CRISPR/Cas9 Delivery in Different Tumors

### Lung cancer

**Fig. 4.**
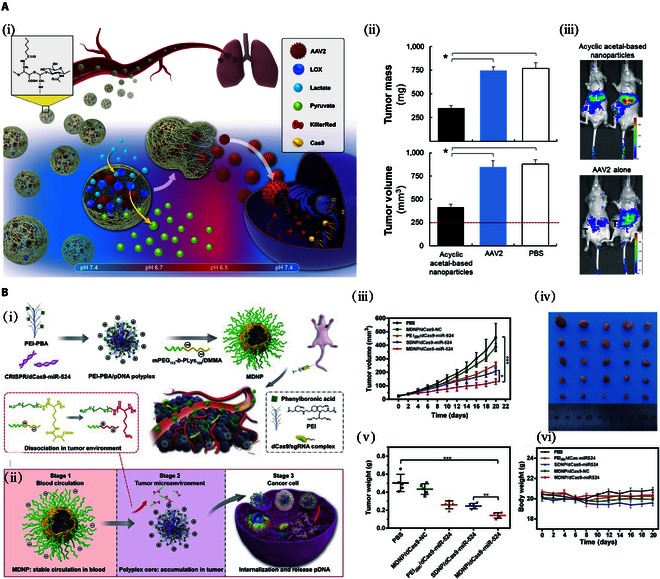
Applications of biomaterial-based CRISPR/Cas9 delivery in lung cancer and breast cancer. (A) An acid-degradable, lactate-oxidizing nanoparticle formulation for non-small cell lung cancer virotherapy. (i) Schematic diagram illustrating acyclic acetal-based nanoparticles for the CRISPR/Cas9 delivery system. Under different treatment strategies, (ii) comparison of tumor weight and volume in mice in each group and (iii) representative in vivo images of luminescence shown in the livers and tumors of mice on day 7 of treatment (T: tumor; L: liver) [171]. Copyright 2022, S.-Ja Tseng et al. (B) A multistage delivery nanoparticle (MDNP) that enables tumor-targeted delivery of the CRISPR/dCas9 system has good antitumor effects in human breast cancer cells. (i) Schematic illustration of the preparation of MDNP and delivery process after intravenous injection; (ii) multistage delivery of the CRISPR/dCas9 system from blood circulation to tumor cells via MDNP. (iii and iv) Tumor volume and (v) weight under different treatment strategies; (vi) body weight of mice under different treatment strategies [184]. Copyright 2022, Qi Liu et al.

The high mortality rate of lung cancer is associated with its susceptibility to develop resistance to targeted drugs. The development of drug resistance in lung cancer is related to continuous gene mutation, and this mutation may be dependent on the production of lactic acid. Therefore, strategies to deplete or oxidize lactate in combination with currently available therapies have emerged for the treatment of lung cancer [170]. Tseng et al. used open-ringing of oxidized hyaluronic acid (HA) conjugated with recombinant adeno-associated virus serotype 2 (AAV2), lactate oxidase, and hexanoamide to assemble acyclic acetal-based nanoparticles. This nanoparticle can consume lactate in the extracellular microenvironment of lung cancer cells, resulting in a local decrease in pH. Since the AAV viral capsid has a pH-sensitive protease, a lower pH value could facilitate internalization of the virus into cells to improve AAV viral transduction. The findings suggest that viral therapies improve the efficacy of viral therapies by delivering AAV2 in vitro and in vivo lung cancer models that exhibit resistance to EGFR-tyrosine kinase inhibitors. The nanoparticle responds to lactate secreted by metabolically reprogrammed lung cancer cells and activates the oxidation of lactate to pyruvate, resulting in the dissolution of acyclic acetal-based nanoparticles. In addition, viral transduction is enhanced, leading to more effective treatments (Fig. 4A) [171]. Noureddine et al. introduced a lipid-encapsulated MSN (LC-MSN) delivery vector loaded with CRISPR components to cancer cells. The LC-MSN vector yielded good gene editing efficiency and anti-cancer effects in A549 lung cancer cells [157]. Liu et al. used protamine sulfate (PS), which contains an NLS, to concentrate plasmid DNA and promote nuclear-targeted delivery. The use of liposomes to encapsulate protein/DNA complexes prevented nuclease degradation in the blood circulation. The PS@Lip/pCas9 vector was further modified with distearyl phosphate ethanolamine-PEG-HA to allow for active targeting of tumor cells. The authors showed that the modified vector delivered the CRISPR/Cas9 plasmid. Knockdown of the mutT homolog1 gene with the modified vector PS@Lip/pCas9 promoted tumor cell apoptosis, and reduced liver metastasis [172]. Kim et al. used Cas9 fused with low-molecular-weight protamine and an NLS to form a triple complex with crRNA/tracrRNA. The transmembrane peptide protamine and NLS can mediate the entry of the complex into the cell and nucleus. Intratumoral injection of the ternary complex can disrupt KRAS and inhibit the growth of cancer cells [173].

### Liver cancer

In 2020, new primary liver cancer cases accounted for 4.7% and the death cases accounted for 8.3%. It has the third highest mortality rate among all kinds of tumors [1]. The liver has rich blood flow, and it is easy to obtain and accumulate nanocarriers. Therefore, vector delivery of genes to treat liver cancer is a promising treatment [174]. Synthetic nonviral delivery systems can cause a degree of immunogenicity and hepatotoxicity, but naturally occurring endogenous vectors can avoid these problems. Wan et al. used hepatic stellate cell-derived exosomes to load Cas9–sgRNA complexes using electric shock. After administration through the tail vein in mice, exosomes targeted KAT5 in the liver and had a significant inhibitory effect on liver cancer [175]. Nie et al. developed an unlockable nanocomposite with a negatively charged heparin core and a positively charged cyclodextrin-PGEA shell (Hep@PGEA) and applied it to the treatment of hepatocellular carcinoma. The study found that the Hep@PGEA/pCas9 system showed high antitumor efficiency by inducing apoptosis and inhibiting the proliferation, migration, and invasion of liver cancer cells. The application of Hep@PGEA/pCas9 in a mouse model of orthotopic liver cancer showed significant accumulation in the liver of mice and achieved significant antitumor effects. The Hep@PGEA/pCas9 system has also shown significant improvement in the treatment of liver cancer with sorafenib and offers promising potential for combination liver cancer therapy [176]. Qi et al. used an amino-epoxide ring-opening reaction to construct a lactose-based reduction-responsive cationic gene vector (LBP). The LBP-mediated CRIPSR/Cas9 system enhanced the antitumor effect of anti-cancer drugs and accelerated liver tumor cell apoptosis [177].

### Pancreatic cancer

Pancreatic cancer is a highly aggressive cancer with a poor prognosis [178]. Li et al. successfully constructed plasmids encoding Cas9 and sgRNA targeting HIF-1α and co-encapsulated with PTX in an R8-dGR peptide-modified cationic liposome (R8-dGR-Lip). R8-dGR-Lip has high tumor cell targeting and permeability to tumor tissues. The study found that R8-dGR-Lip/PTX/pHIF-1α successfully down-regulated HIF-1α and its downstream molecules, VEGF and MMP-9. Inhibition of HIF-1α can enhance the antitumor and anti-metastatic effects of PTX in the BxPC-3 pancreatic tumor model. In addition, HIF-1α blockade also promotes the cytotoxicity of PTX to pancreatic cancer cell lines [179]. Tao et al. constructed a novel nanocarrier platform (AP-RNP-F) consisting of a multibranched gold nanooctopus core (AuNO) and a mesoporous polydopamine shell (mPDAs). The shell is loaded with CRISPR-Cas9 RNP, coated with PEG-folate (PEG-FA). Multibranched gold nanostructures exhibit localized surface plasmon resonance, excellent tissue penetration, and high photothermal conversion efficiency. Because PEG-FA prolongs blood circulation and imparts active targeting, AP-RNP-F can be efficiently delivered into tumor cells. When AP-RNP-F was endocytosed, acidic environment stimulation and NIR irradiation could precisely trigger the release of AP-RNP-F-loaded Cas9 RNPs into the nucleus. In pancreatic cancer cells, knocking down HSP9α by Cas9 RNP made the tumor cells more sensitive to the thermal effect of AuNO@mPD under NIR-II irradiation, leading to apoptosis of tumor cells without affecting the state of surrounding normal cells [180].

### Breast cancer

Breast cancer treatment has progressed steadily and effective therapeutic targets have been identified for some subtypes. For example, anti-estrogen endocrine therapy is effective for treating luminal breast cancer and anti-HER2-targeted therapy works well for HER2-positive breast cancer. However, TNBC still lacks clear therapeutic targets [181]. Many studies have used the CRISPR/Cas9 system for the diagnosis and treatment of breast cancer [182]. In developing a treatment for TNBC, Guo et al. encapsulated the CRISPR/Cas9 in a soft nanogel consisting of non-toxic fat molecules and hydrogels. Antibodies were attached to the gel surface to guide CRISPR nanoparticles to the tumor site by recognizing and locating ICAM-1, a possible drug target for TNBC. The delivery success rate was 81%, and prevented tumor growth in 77% of TNBC mice [167]. Li et al. constructed a proton-activated DNA nanosystem (H-DNC) that efficiently delivers DNases and Cas9/sgRNA RNPs in vivo. Specifically, an ultra-long ssDNA strand containing the sgRNA recognition sequence of sgRNA in Cas9/sgRNA, DNAzyme sequence, and Hhal enzyme cleavage site was synthesized as the scaffold of the nanosystem. The DNAzyme cofactor Mn^2+^ was used to compress DNA chains to form nanoparticles, and acid-degradable polymer-coated Hhal enzymes were assembled on the surface of nanoparticles. After H-DNC accumulates at the tumor site, H-DNC is readily internalized into cancer cells via lysosome-mediated pathways. In the acidic environment of lysosomes, the polymer coating on Hhal is degraded, and no additional enzyme injection is required, allowing for the automatic release of DNAzyme and Cas9/sgRNA RNPs. After the application of this system, the apoptosis rate is high, and the combination treatment of breast cancer can be realized [183]. Liu et al. used 2,3-dimethylmaleic acid (DMMA) to modify PEG-b-polylysine to obtain mPEG-b-PLys/DMMA, coated with phenylboric acid (PBA)-PEI to obtain weakly acidic microenvironment-responsive core–shell structured nanoparticles(MDNP). The microenvironment-responsive polymer shell gives MDNPs the ability to exhibit different surface properties at different stages of delivery, enabling MNDPs to overcome multiple physiological barriers and deliver payloads to tumor tissues with optimal efficiency. In an acidic environment, DMMA degrades and PBA enhances cellular uptake by binding to sialic acid on the tumor surface. Systemic administration of MDNP/dCas9-miR-524 to breast cancer model mice achieved effective up-regulation of miR-524 in tumors, resulting in simultaneous interference of multiple signaling pathways related to cancer cell proliferation and significant tumor growth retardation (Fig. 4B) [184].

### Ovarian cancer

LNPs were used to deliver CRISPR, which edited PLK1 in tumor cells with 82% efficiency and improved the overall survival of mice by approximately 80% [124]. Kim et al. reported that cancer exosomes selectively accumulated in ovarian tumors of mice, providing an effective in vivo delivery route. CRISPR/Cas9-loaded exosomes inhibited PARP expression and induced apoptosis in ovarian cancer cells [185]. Studies have shown that certain surface receptors are commonly overexpressed in tumors, and the use of nanoparticles targeting these receptors as a vector is a strategy [186]. Lu et al. successfully designed a reduction-sensitive fluorinated Pt (IV) universal transfection nanoplatform (PtUTP-F) by reducing Pt (IV) prodrug and fluorinated low-molecular-weight polyethylenimine (PEI1.8K-F) (Mw = 1,800) and lysine diisocyanate, which were further protected by the PEG side chain in a stepwise manner. It was found that PtUTP-F had excellent cell uptake, endosome/lysosomal escape and gene release, and high transfection efficiency, which could efficiently transfect a variety of cells. PtUTP-F/dCas9-CT45 has been validated to produce CRISPR activation of CT45 expression, thereby decreasing protein phosphatase 4C (PP4C) activity and blocking platinum-induced DNA repair pathways, thereby promoting sensitivity to anti-cancer Pt (II) drugs, enabling synergistic and personalized treatment of ovarian cancer in vitro and in vivo [187].

### Cervical cancer

**Fig. 5.**
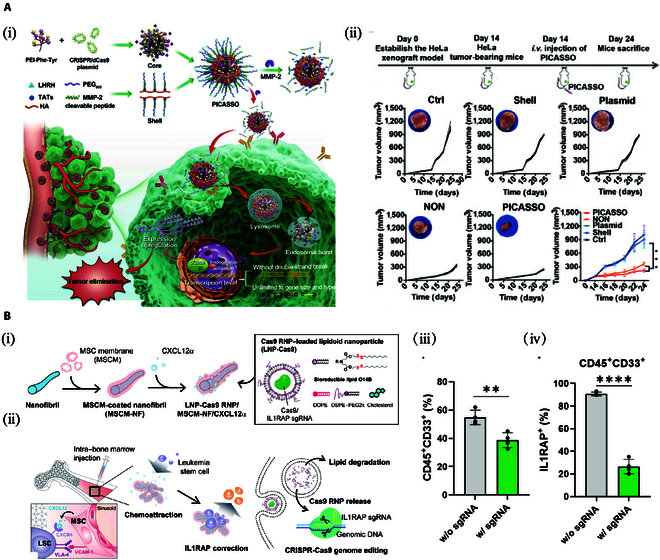
Applications of biomaterial-based CRISPR/Cas9 delivery in cervical cancer and acute myeloid leukemia. (A) A programmable hierarchical-responsive nanoCRISPR (PICASSO) for cervical cancer therapy. (i) Schematic illustration of the IPICASSO delivering CRISPR/cas9 plasmid to exert gene editing in human cervical cancer cells. (ii) Individual tumor growth profiles and average tumor volume of mice treated with various agents [188]. Copyright 2021, Chao Liu et al. (B) Mesenchymal stem cell membrane-coated nanofibril scaffold-mediated CRISPR/Cas9 system delivery for acute myeloid leukemia therapy. (i) Assembly of MSCM-NF and delivery of CRISPR/Cas9. (ii) Schematic diagram and mechanism of MSCM-NF for treatment. (iii) Percentage of human CD45CD33 population and (iv) human IL1RAP expression in CD45 and CD33 population in the bone marrow harvested from recipient mice [192]. Copyright 2021, Tzu-Chieh Ho et al.

A programmed graded response CRISPR platform (PICASSO) by self-assembly using a multifunctional polymer shell and amino acid-modified core was efficiently loaded with CRISPR/dCas9 plasmids. Specifically, an amino acid-modified polyethylenimine was synthesized by modifying PEI 1.8K with phenylalanine (Phe) and tyrosine (Tyr) (PPT, the core of PICASSO). PPT35 (the grafting ratio of Phe and Tyr was 35%) was selected to construct multiple complexes (core@CRISPRa) due to its good transfection efficiency in previous experiments. Then, PICASSO multifunctional shell (LHRH polypeptide ligand-PEG600-MMP-2-cleavable adaptor-TATS-HA) was synthesized. The PICASSOs were obtained by coating the shell onto the core@CRISPRa polyplex by electrostatic interaction. PICASSO can accumulate at the tumor site through the prolonged circulation of blood and, at the same time, can escape capture and degradation by lysosomes in the cell and release the CRISPR/dCas9 system. When the TRAIL gene was selected as a model therapeutic target, the PICASSO platform activated endogenous TRAIL gene activation in tumor-bearing mice and efficiently induced TRAIL gene expression and apoptosis in human HeLa cervical cancer cells (Fig. 5A) [188].

### Melanoma

Serial mutations are the main cause of melanoma progression [189]. In recent years, oncogene-targeting agents and immune checkpoint inhibitors have been used to treat patients with melanoma and have been shown to improve overall patient survival; however, the use of these drugs can cause toxicity and resistance in some patients [190]. Gene therapy is a good alternative for melanoma treatment. Wang et al. combined lipids and AuNPs, 2 separate carriers for gene delivery, to develop a multifunctional system. They employed sgRNAs targeting the Plk-1 gene. The Cas9-sgPlk1 plasmid (CP) was loaded onto AuNPs using TAT polypeptide to form AuNP/CP (ACPs), which were then coated with lipids and DSPE-PEG2000 to form lipid-coated ACPs (LACPs). LACPs successfully achieved Plk-1 gene knockdown, resulting in effective melanoma therapy [119]. Lee et al. developed the NanoRNP system as a novel approach to melanin immunotherapy. Their chemical derivatization of Cas9 with the polymer allows the protein to be firmly bound to sgRNA into condensed RNPs, which form nanoassembled structures by further complexing with pso. In addition to forming stable and condensed complexes, Cas9 conjugated to low-molecular-weight polymers is able to interact strongly with target cell membranes, which will promote the internalization of RNPs into cells. The study found that delivery of NanoRNP into B16 melanoma cells leads to efficient internalization and gene disruption with low cytotoxicity, leading to the persistence of PD-L1. In vivo delivery in a mouse melanoma model has shown that NanoRNP can induce indels in tumor target cells with high frequency, resulting in major suppression of tumor growth, without involving combination therapy. In addition, NanoRNP blocks PD-L1 checkpoints in tumor tissues and promotes T cell infiltration and effector cytokine release, which are characteristic of activating antitumor immunity and suppressing immunosuppressive myeloid cells [191].

### Other cancers

Acute myeloid leukemia (AML), characterized by abnormal proliferation and impaired differentiation, is the main type of acute leukemia in adults. A bioreducible reduced liposome-encapsulated Cas9/sgRNA RNP (LNP-Cas9 RNP) was used to target interleukin-1 receptor accessory protein (IL1RAP), which is a key player in human leukemia stem cells (LSCs). To optimize bioreducible LNPs targeting LSCs, the authors synthesized and tested 8 bioreducible lipids. The Cas9/IL1RAP-sgRNA complex (Cas9-RNP) is coated by LNPs by self-assembly and is used to treat the human leukemia cell line expressing IL1RAP. Liposomes that show the highest gene editing efficiency in leukemia cells were selected for further study. LNP-Cas9 RNP and the chemokine CXCL12α were loaded onto a mesenchymal stem cell membrane-coated nanofibril (MSCM-NF) scaffold mimicking the bone marrow microenvironment. CXCL12α release induced LSC migration to the MSCM-NF scaffold in vitro and LNP-Cas9 RNP efficiently edited IL1RAP, resulting in IL1RAP knockout. This NF scaffold-mediated CRISPR-Cas9 delivery system provides a safe and effective Cas9 delivery platform for targeting LSCs to improve AML treatments (Fig. 5B) [192]. Zou et al. modified Angiopep-2, a ligand capable of binding LRP-1 protein, on a thin, disulfide-linked PEG polymer shell. LRP-1 is highly expressed in blood–brain barrier endothelial cells and in glioblastoma (GBM). The modified polymer shell encapsulated the Cas9–sgRNA RNP complex into nanocapsules with a nearly neutral surface charge, which protected the internal therapeutic components from ribonuclease degradation and promoted blood stability, thereby enhancing blood–brain barrier permeability. Nanocapsules can transport CRISPR/Cas9 across the blood–brain barrier to target brain lesions and effectively edit the oncogene PLK1. The editing efficiency of PLK1 was as high as 38.1%, which significantly reduced PLK1 expression and inhibited cell division in GBM. The median survival time of mice was prolonged by nearly 3-fold after treatment with the nanocapsules [133]. Ruan et al. developed a brain targeting CRISPR/Cas9-based nanodrugs to treat human GBM in situ. The formulated nanoparticles are denoted as Ang-NP@RNP and were prepared by complexation between angiopep-2 decorated poly(ethylene glycol)-block-poly(N-(3-methacrylamidopropyl) guanidinium) (Ang-PEG-b-PGu)/poly(ethylene glycol)-block-poly[N-(3-methacrylamidopropyl) guanidinium-co-2,2,3,3-tetrafluoropropyl methacrylate] (PEG-b-P(GuF)) mixture and Cas9/gRNA RNP. Studies have found that Ang-NP@RN has the characteristics of long blood circulation time, high blood–brain barrier penetration, and high gene editing efficiency in vitro and in vivo, which can effectively inhibit tumor growth. Ang-NP@RNP significantly improved the median survival time to 40 days in mice with GBM in situ with no significant side effects or off-target effects [193]. Wan et al. designed an efficient supramolecular polymer (CP/Ad-SS-GD) for the delivery of Cas9 RNP. Specifically, the host chain segment (CD-β-PEI (CP)) was composed of β-cyclodextrin-conjugated low-molecular-weight PEI, and the guest fragment was composed of biguanide groups and adamantane (Ad-SS-GD), CP, and Ad-SS-GD guanidinylation of CP through supramolecular complexation to generate CP/Ad-SS-GD. It was found that CP/AD-SS-GD could effectively promote the delivery of Cas9 RNP to CRC cells and easily release Cas9 RNP. In addition, the CP/Ad-SS-GD/RNP nanocomplex has the ability to target mutant KRAS, resulting in potent inhibition of tumor cell proliferation and apoptosis in vitro. By decorating with HA, nanocomplexes targeting mutant KRAS exhibit excellent anticancer efficacy in colorectal cancer (CRC) xenograft models and effectively inhibit metastasis in vivo with very low systemic toxicity [194]. Cheng et al. used an amphiphilic PEI derivative to deliver pCas9/sgRNA specific for PD-L1. The resulting albumin-coated polyamylose was shown to reduce PD-L1 gene expression in CT26 CRC cells, with a greater than 6-fold reduction in PD-L1 protein levels compared to treatment with lipofuscin-like contaminants and genome-editing components [195]. Zhang et al. used an aptamer that can recognize the surface of tumor cells as a targeting ligand coupled with CRISPR/Cas9-packaged polymeric biomaterials (PEG-PEI-Chol) to form a novel delivery system. This delivery system promoted the entry of CRISPR/Cas9 into osteosarcoma cells and inhibited the production and secretion of VEGFA in vitro. In vivo, CRISPR/Cas9 delivery accelerated the specific aggregation of osteosarcoma and lung metastatic lesions in a mouse model, inhibited tumor deterioration, and reduced lung metastasis [196].

## Conclusions and Prospects

The CRISPR/Cas9 gene editing technology has had a tremendous impact in the field of gene therapy. The CRISPR/Cas9 system is widely used to regulate gene expression, auxiliary disease diagnosis, collaborate immunotherapy, and develop anticancer drugs, and construct models, providing new hope for tumor therapy [197]. The CRISPR/Cas9 system has been used to treat a variety of cancers, such as lung cancer, pancreatic cancer, liver cancer, breast cancer, ovarian cancer, melanoma, and glioblastoma, and has achieved significant therapeutic effects. However, many challenges remain for CRISPR/Cas9, such as low delivery efficiency, off-target effects, and detrimental immune responses. A safe and efficient delivery system is the prerequisite for the clinical application of the CRISPR/Cas9 system. There are many ways to deliver the CRISPR/Cas9 system. While physical delivery approaches are not applicable in vivo and viral vectors are susceptible to high mutation rates and present carcinogenic risks, the use of non-viral delivery vectors shows promise. Owing to their high packaging capacity, low immunogenicity, and ease of assembly, non-viral vectors are the most suitable delivery modality for in vivo drug delivery. In recent years, biological materials have become the first choice for CRISPR/Cas9 non-viral vectors because of their high gene editing efficiency, high tissue/cell specificity, and low immunogenicity. The successful application of various biomaterials in CRISPR/Cas9 delivery systems demonstrates their versatility and adjustability, making them an attractive option to address many biological questions related to CRISPR/Cas9 delivery.

Although biomaterials have many advantages, there are still several obstacles in the delivery of CRISPR/Cas9 systems. (a) The safety and biocompatibility of biomaterials. Most biomaterials, such as cationic liposomes and polymers, may have a certain degree of toxicity or inflammatory reaction to normal cells, affecting their physiological functions. Biomaterials are difficult to degrade and excrete in vivo, which will lead to their accumulation in normal tissues and increase the damage to normal tissues. Therefore, it is still necessary to explore better surface modification methods to reduce the toxicity of materials, and effectively improve the biodegradability and compatibility of materials. In addition, it is also necessary to formulate relevant international regulatory standards and reliable methods to evaluate the safety of biomaterial. (b) Low delivery efficiency and poor targeting. As an exogenous substance, unmodified biomaterials are susceptible to phagocytosis by macrophages in the reticuloendothelial system. Furthermore, there is a complex tumor microenvironment, and tumor tissue has characteristics such as high vascular density, low maturity, disordered branching, highly irregular distribution, and isomerization. These obstacles increase the difficulty and complexity of targeted delivery of biomaterials to the CRISPR/Cas9 system. In addition, the EPR effect is highly heterogeneous, and the physical and chemical properties of biomaterials such as size and charge also affect its delivery efficiency. Therefore, when designing biomaterials, adjusting the physical and chemical properties of the carrier, such as particle size, shape, and surface charge, and making specific modifications will help to improve its tumor-targeting capability and increase delivery efficiency. (c) Safety of clinical transformation. At present, most of the experiments on biomaterials are only conducted in vitro and involve animals. When biomaterials are applied to clinical treatment, their potential toxicity should be fully considered, such as inflammatory release against cells, genome integration, and proto-oncogene activation. These problems need to be fully verified and optimized in in vitro experiments and animal models to ensure good safety and efficacy in human bodies before they can be considered for clinical transformation. (d) The preparation process of biomaterials is complex and difficult to produce on a large scale. The related research on biomaterials is mainly used in drug development. There is relatively little research on the equipment, quality control, and cost control needed for the large-scale production of biomaterials. The problems faced, such as the high cost of metal nanoparticles and the complicated preparation process of safe and effective delivery carriers, will hinder the clinical application of biomaterials. In addition, based on the toxicity and safety problems of materials and sizes, long-term biocompatibility, cycle half-life, repeatability, and serum stability, anyone may destroy the biomaterial pharmaceutical process. Producing safe and effective biomaterials in the delivery of CRISPR/Cas9 for tumor treatment on a large scale under cost control conditions will be a great challenge.

In the future, there are still many directions that need to be explored for the application of biomaterials as delivery vectors for the CRISPR/Cas9 system in clinical tumor treatment, namely, continuously modifying and improving biomaterials to maximize targeted delivery efficiency, increasing the degradation of biomaterials to reduce accumulated toxicity in the body, prolonging blood circulation time, preventing rapid clearance by the kidneys and phagocytosis by the liver or spleen, achieving effective encapsulation and protection of RNA during delivery, avoiding degradation based on nucleases, optimizing preparation processes, and reducing costs. With the emergence of new biomaterial technologies and our growing understanding of the biological challenges associated with CRISPR/Cas9 delivery, it is expected that biomaterials will ultimately be applied in clinical tumor treatment.
